# MSTA-SlowFast: A Student Behavior Detector for Classroom Environments

**DOI:** 10.3390/s23115205

**Published:** 2023-05-30

**Authors:** Shiwen Zhang, Hong Liu, Cheng Sun, Xingjin Wu, Pei Wen, Fei Yu, Jin Zhang

**Affiliations:** 1 College of Information Science and Engineering, Hunan Normal University, Changsha 410081, China; 2 School of Mathematics and Statistics, Hunan Normal University, Changsha 410081, China; 3 School of Computer and Communication Engineering, Changsha University of Science & Technology, Changsha 410114, China

**Keywords:** classroom behavior detection, behavior detection, SlowFast model, attention mechanism

## Abstract

Detecting students’ classroom behaviors from instructional videos is important for instructional assessment, analyzing students’ learning status, and improving teaching quality. To achieve effective detection of student classroom behavior based on videos, this paper proposes a classroom behavior detection model based on the improved SlowFast. First, a Multi-scale Spatial-Temporal Attention (MSTA) module is added to SlowFast to improve the ability of the model to extract multi-scale spatial and temporal information in the feature maps. Second, Efficient Temporal Attention (ETA) is introduced to make the model more focused on the salient features of the behavior in the temporal domain. Finally, a spatio-temporal-oriented student classroom behavior dataset is constructed. The experimental results show that, compared with SlowFast, our proposed MSTA-SlowFast has a better detection performance with mean average precision (mAP) improvement of 5.63% on the self-made classroom behavior detection dataset.

## 1. Introduction

Intelligent education has become one of the inevitable trends in the future development of education [1]. The classroom is an important part of building intelligent schools. When evaluating the quality of classroom teaching, students’ classroom behavior can be used as important reference content. Students’ classroom behaviors can reflect students’ learning state well [2]. At the same time, the behaviors in the recorded teaching videos can be analyzed accordingly after class, which can help teachers to adjust teaching methods and progress in time to achieve better teaching results.

In traditional classrooms, teachers need to observe students’ classroom behavior manually. However, this approach cannot attend to all students at the same time, makes it difficult to form timely and effective feedback, and brings a certain burden on teachers’ teaching work. With the increasing sophistication of artificial intelligence, the detection of student behavior in the classroom through deep learning and computer-vision-enabled techniques is gaining attention [3]. The use of computer-assisted instruction and the automated detecting and analyzing of student behavior in the classroom has also become a research hotspot in smart education [4,5,6].

Classroom behavior detection is generally divided into approaches based on object detection [7], pose recognition [8], and video behavior recognition or detection [9]. With growing advances in video behavior detection technology, classroom behavior detection based on instructional videos has become possible. In the field of video behavior identification, deep learning’s ongoing development has produced some excellent outcomes. Among them, SlowFast [10] achieves good detection results in Kinetics [11] and Charades [12] behavior recognition datasets, and AVA (Atomic Visual Actions) [13] spatio-temporal behavior detection dataset. SlowFast also has great application scenarios in real-world problems. For example, Cui et al. [14] combined SlowFast with a bounding box labeling algorithm to detect the smoke phenomenon in a forest. Li et al. [15] applied SlowFast to a pig behavior recognition scenario. Joshi et al. [16] used SlowFast ResNet-50 to detect abnormal behavior in a surveillance system. In this study, the issue of detecting student behavior in a classroom setting is addressed using SlowFast.

The classroom scenario is complex, with masking between students and the need to detect the behavior of multiple people at the same time. To improve the detection accuracy, this paper proposes an improved SlowFast network for classroom behavior detection to perform multi-label detection of common classroom behaviors of students in videos. The model focuses on detecting seven common classroom behaviors, such as looking at the board, raising hands, lying on the table, talking, and bowing heads, and finally outputting information about students’ positions and behaviors. Different classroom behaviors reflect students’ learning status and concentration. For example, when students show negative behaviors such as sleeping and turning around, they are generally inattentive or confused about the teaching content. When students look at the blackboard carefully and raise their hands to answer questions, it means that they are interested in the content taught by the teacher. Detecting these behaviors can help analyze students’ participation and concentration, so as to assess the effectiveness of the classroom, help teachers understand students’ learning in the classroom, and help to adjust the teaching schedule and improve teaching methods in time. Additionally, to validate the proposed classroom behavior detection algorithm, we constructed a students’ classroom behavior dataset. The primary contributions are as follows:

- Classroom instructional videos were collected to mark common student behaviors in the classroom, and a classroom behavior dataset was constructed as a basis for detecting student behaviors.
- A student behavior detection model based on an improved SlowFast network was proposed. The model’s ability to acquire spatial, channel, and temporal features was improved, and the detection accuracy was increased, with the introduction of Multi-Scale Spatial-Temporal Attention (MSTA) and Efficient Temporal Attention (ETA) modules.
- Finally, to verify the effectiveness of the revised approach, experiments were carried out. The findings showed a significant improvement in the improved model’s mean Average Precision (mAP), which could be utilized to detect classroom conduct.

## 2. Related Work

### 2.1. Video Behavior Detection

Mainstream behavior detection algorithms can be generally classified into behavior recognition, temporal behavior detection, and spatio-temporal behavior detection. Among them, behavior recognition mainly identifies the category of behavior. Temporal behavior detection identifies the time period in which the behavior in the video occurs and determines the category of the behavior in the video. Spatio-temporal behavior detection focuses on identifying the coordinate position of the person in the video and identifying the duration of the person’s behavior with the category of the behavior. In this paper, classroom behavior detection focuses on the location and category of classroom behavior occurrence, so spatio-temporal behavior detection is used for this purpose.

For the problem of spatio-temporal feature extraction in the field of video behavior understanding, researchers had already proposed many effective backbone network structures. For example, 3D convolutional neural networks (C3D) [17] use three-dimensional convolution to extract the spatio-temporal features of actions, which can identify actions more accurately. Karen et al. [18] proposed a dual-stream network, where one pathway extracts spatial features through RGB images while the other pathway extracts temporal features through optical flow images.

With the proposed AVA [13] for the Atomic Vision Action Video dataset, the focus of the spatio-temporal behavior detection task has gradually shifted toward behavioral interactions, and many behavior detection algorithms for this dataset have emerged. Christoph et al. [10] proposed the SlowFast network, based on 3D convolution, to obtain behavioral features. The network has performed well in both behavior recognition and behavior detection tasks. It consists of two pathways with distinct temporal rates that are responsible for the acquisition of spatial and temporal information, respectively. In place of the double-branch approach, Christoph [19] presented an extended 3D convolutional network (X3D), which gradually modifies the model’s width parameter to require less computational work while producing superior results. Li et al. [20] analyzed the effect of time dependence on behavior detection by placing the behavior detection in a Long-Short Term Context (LSTC).

Meanwhile, a number of researchers have suggested new spatiotemporal detection methods. Okan et al. [21] proposed a new spatio-temporal behavior detection framework, named YOWO (You Only Watch Once), which is well suited for real-time spatio-temporal behavior detection in videos because it integrates temporal and spatial information into the framework and uses only one network to directly extract both. Fan et al. [22] proposed an MViT (Multiscale Vision Transformers) model for video and image recognition by combining multi-scale feature pyramid structures to achieve the extraction of video features at different levels, and encoding the features using Transformer to enable the model to better understand the visual content. Bertasius et al. [23] proposed a new detection network, TimeSformer, implemented by a convolution-free approach, which employs a self-attentive module instead of convolution.

### 2.2. Behavior Detection in Classroom Scenarios

Classroom scenarios with severe occlusion and numerous student targets pose a great challenge for classroom behavior detection. Recently, computer vision, target detection, and image classification techniques have also been applied to classroom behavior detection tasks.

By employing object detection to identify classroom behavior, the behavior that needs to be identified is treated directly as a target object, and the network is then utilized to extract spatial features to identify the behavior. Liu et al. [24] used the YOLOv3 algorithm for student anomalous behavior recognition with the addition of RFB and SE-Res2net modules to improve the model for small target and crowd occlusion problems in the classroom environment. Tang et al. [25] performed classroom behavior detection based on pictures, adding a feature pyramid structure and an attention mechanism to the YOLOv5 classroom behavior detection model to address the problem of high occlusion in the classroom environment.

Pose recognition is usually used to identify human behavior by using localized human key point detection. Lin et al. [26] used the OpenPose framework to collect skeletal information from students and classify the extracted skeletal information into behaviors by means of a neural network. Yu et al. [27] collected classroom data using the Microsoft Kinet device for face recognition and then collected human skeleton information to extract features for behavior classification.

Recently, some researchers have implemented classroom behavior detection through video behavior detection techniques; Huang et al. [28] proposed a deep spatio-temporal residual convolutional neural network, and combined target detection and target tracking algorithms to detect the classroom behaviors of multiple students in teaching videos in real-time, and achieved good operational results. To realize real-time recognition of classroom behaviors for multi-student objectives, Xiao et al. [29] used the YOLOX algorithm to extract the student behavior at a moment in the instructional video and used CNN (Convolutional Neural Network) to learn the spatio-temporal information.

The object-based detection approach ignores the temporal characteristics of the behavior and cannot combine contextual semantic information. The human keypoint-based behavior detection is more computationally intensive and has stricter scene requirements, resulting in its poor stability in different scenes. Video-based behavior detection can capture the action information of behavior more comprehensively and achieve more accurate detection of behavior, but the computational effort also increases. Meanwhile, the above study found that classroom behavior detection has certain shortcomings [25]. First, there are relatively few publicly available classroom scenario datasets. Second, some of the algorithms are only capable of detecting a single behavior detection target at the same time, so they cannot be used in classroom scenarios where the task of behavior recognition is performed on multiple students at the same time.

## 3. Methods

**Figure 1:**
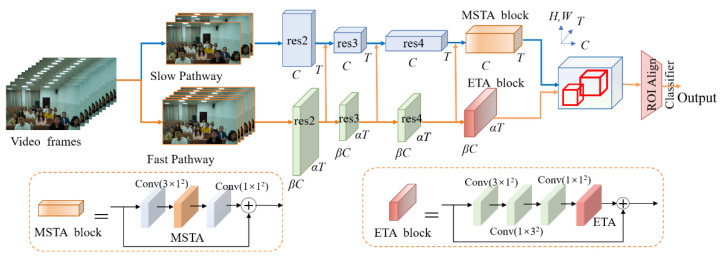
MSTA-SlowFast model structure.

The SlowFast algorithm has made certain research progression in behavior detection, but its detection accuracy is still lacking in the classroom environment, and the accuracy rate is not high for actions with a small sample size and is more difficult to identify. Therefore, on the basis of a SlowFast network, firstly, an MSTA (Multi-scale Spatial-Temporal Attention) module is introduced into the Slow path to effectively extract multi-scale spatial information, establish remote channel dependence, and add temporal attention. Secondly, the ETA (Efficient Temporal Attention) module for temporal dimension is introduced into the Fast pathway to effectively calculate temporal attention and strengthen the ability to perceive temporal features of actions. Figure 1 shows the structure of the modified MSTA-SlowFast model.

### 3.1. SlowFast Network

The SlowFast network is a dual-stream network based on the 3D CNN model, which includes two pathways. The Slow pathway mainly acquires spatial semantic information by using a 3D CNN model with a low frame rate. Additionally, the Fast pathway mainly acquires action information using a high-frame-rate 3D CNN model, but with a smaller convolution width and less number of channels. Meanwhile, the different spatio-temporal features are fused by lateral connections. Both paths have a 3D ResNet [30] network structure.

The SlowFast network settings include τ, α, and β parameters, which represent the video sampling step, the frame rate ratio of the two pathways, and their channel number ratio, respectively. Specifically, the Slow pathway to Fast pathway frame-rate ratio is 1:α (α>1) and the channel number ratio is 1:β (β<1). The Fast pathway weakens its ability to process spatial information by using smaller convolutions and fewer channels, thus reducing the computational effort and improving its expressiveness in the time domain.

The network fuses the features extracted from the Fast pathway into the Slow pathway through multiple lateral connections. Generally, the feature maps of the Fast pathway output are converted from $\left\{\alpha T,\;S^{2},\beta C\right\}$ to $\left\{T,\;S^{2},\;\alpha \beta C\right\}$ by using time dimensional convolution, and then fused with the feature maps of size $\left\{T,\;S^{2},C\right\}$ of the Slow pathway.

The model needs to detect the student position in the key frame by the detector during the detection and pass the detection result into the network, and faster R-CNN [31] is used as the human detector in this paper. The network finally calculates the RoI (region-of-interest) features through the RoIAlign algorithm and sends them to the multi-label classification prediction based on Sigmoid.

### 3.2. MSTA Module

**Figure 2:**
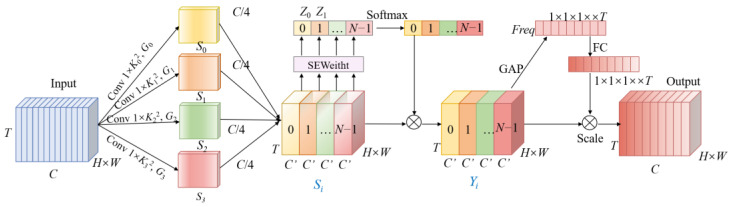
MSTA module structure.

The model typically uses the attention mechanism to pick out more crucial details and concentrate more on important areas of the image. A SENet (Squeeze-and-Excitation Network) [32] uses a channel attention mechanism, and each channel’s weight was then adaptively calculated using a fully connected layer after being converted to a single value using GAP (Global Average Pooling). However, it ignores the importance of spatial information. A CBAM (Convolutional Block Attention Module) [33] enriches the attention graph by effectively combining spatial and channel attention, and uses GAP and a global maximum pool to enhance feature diversity. However, SlowFast as a 3D CNN not only needs to acquire channel and spatial information but more importantly, to perform behavior recognition by temporal information. Therefore, inspired by [34,35], we construct a Multi-scale Spatial-Temporal Attention (MSTA) module, that can capture and utilize channel, temporal and differently-sized spatial information more effectively, and establish channel and spatial remote dependencies at the same time. Figure 2 depicts the structure of MSTA, that consists of multi-scale spatial feature extraction, channel attention, and temporal attention.

The MSTA module first extracts the multi-scale spatial features, dividing the feature map X into N parts. Each part contains $C^{\prime }$ feature channels, where $C^{\prime }=C/N$. For the division of each channel feature map, multi-scale spatial information is extracted using the 3D convolution of different sizes. The calculation process is shown in Equation (1), where $X_{i}$ denotes the segmented feature map, $\;K_{i}$ denotes the convolutional kernel size, $G_{i}$ denotes the group size, and $G_{i}=2^{(K_{i}-1)/2}$.

(1) $$S_{i}=Conv\left(1\times K_{i}\times K_{i},G_{i}\right)\left(X_{i}\right)\;i=0,1,2,\ldots ,N-1.\;$$

After that, the channel attention weights need to be extracted. The channel weight $Z_{i}$ is calculated by *SEWeight* for different sizes of feature maps $S_{i}$ . After, $Z_{i}$ is rescaled using the Softmax algorithm and then multiplied with the feature map $S_{i}$ of the corresponding scale. The calculation process is shown in Formulas (2) and (3).

(2) $$Z_{i}=SEWeight\left(S_{i}\right)$$

(3) $$Y_{i}=S_{i}⊙Softmax\left(Z_{i}\right)=S_{i}⊙\frac{exp\left(Z_{i}\right)}{\sum_{i=0}^{S-1}exp\left(Z_{i}\right)}$$

Then, the temporal attention weights are calculated by applying them to the feature map Y. Specifically, the overall features in each time dimension are encoded into a global feature t using global pooling. On this basis, the overall feature map is subjected to the excitation operation, that is, the correlation between the temporal dimensions is constructed through two full connection layers and the weights g of the same dimensions are output. The calculation process is shown in Formula (4).

(4) $$g=F_{ex}\left(t,W\right)=\delta \left(g\left(t,W\right)\right)=\delta \left(W_{2}ReLU\left(W_{1}t\right)\right)\;\;$$

Finally, the feature maps are then multiplied by the temporal dimensional weights to provide feature maps with richer multi-scale information. Since the spatial information extracted by the Fast pathway is less, the improvement of the model in this paper is that the MSTA module is introduced in the slow paths, replacing the 1 × 3 × 3 convolution in the middle layer of the res5 residual module.

### 3.3. ETA Module

**Figure 3:**
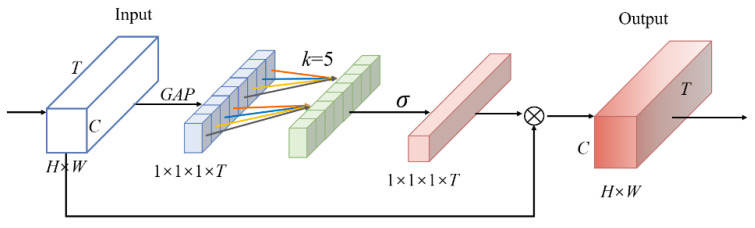
Diagram of the ETA module.

The Fast pathway mainly obtains temporal features of the action and relatively little spatial information. The Efficient Temporal Attention (ETA) module is added to the Fast pathway to enhance model detection performance and help the model better capture action information. The ETA module is built with reference to ECA (Efficient Channel Attention) [36] and uses one-dimensional convolution to efficiently implement local cross-temporal dimensional interactions, avoid dimensionality reduction, and extract temporal channel correlations. Figure 3 shows the structure of the ETA module.

The ETA is calculated as follows: first, the GAP is performed to obtain a 1×1×1×T vector, $X\in R^{W\times H\times T\times C}$. Afterward, the weight of each time dimension is obtained by interacting information across time dimensions. Fast one-dimensional convolution using a convolution kernel of size  k is mostly responsible for achieving this; the formula is as follows:

(5) $$w_{i}=\sigma \left(\sum_{j=1}^{k}w^{j}y_{i}^{j}\right)\;\;\;\;y_{i}^{j}\in \Omega_{i}^{k}\;\;$$

where σ is the Sigmoid function, $y_{i}^{j}$ denotes the feature of the jth adjacent channel of the ith time dimension, and $\Omega_{i}^{k}$ denotes the set of k adjacent channels, where the convolution kernel’s size, k, is derived adaptively by Formula (6). ${\left|t\right|}_{odd}$ denotes the nearest odd number to t.

(6) $$k=\psi \left(T\right)={\left|\frac{\log_{2}T}{\gamma }+\frac{b}{\gamma }\right|}_{odd}$$

The global final objective features are created by multiplying the original feature maps by the weight of the temporal domain. The ETA module avoids dimensionality reduction while taking into consideration the impact of cross-temporal context interactions. In the network, ETA is added to the res5 module of the Fast pathway to enhance the model’s ability to perceive temporal features.

## 4. Experimental Results and Analysis

### 4.1. Dataset

We created a spatiotemporal-oriented classroom student behavior (SCSB) dataset because there are not any publicly accessible classroom datasets that can be used to deal with the issue of video-based classroom behavior detection. Spatiotemporal-oriented behavior detection aims to find the time and space in which the behavior of interest is located from the video and requires multiple frames to be correlated in order to determine the continuous behavior. The dataset is mainly annotated with reference to the publicly available AVA dataset for spatio-temporal behavior detection [13]. The AVA dataset is taken from 437 movies, annotated for 80 categories, and provides temporal labels for one frame per second for bounding boxes and actions.

**Figure 4:**
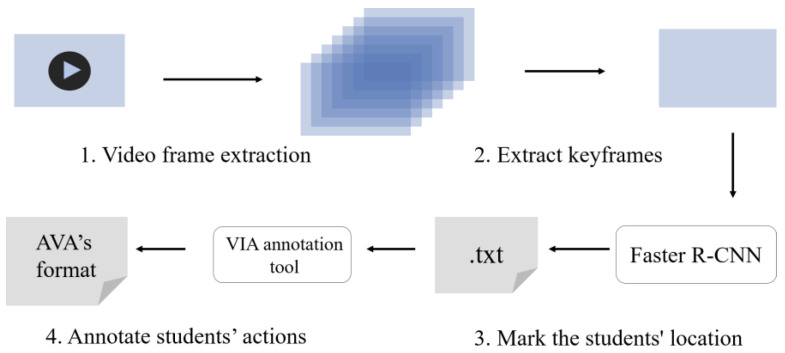
SCSB dataset production process.

Approximately 250 min of classroom instructional videos were filmed in classroom scenes, primarily from the front side of the classroom. The videos were cut and filtered, and more than 600 of them were labeled, each containing 7–20 students, and all were 10 s in length. Seven common classroom behaviors were selected for labeling: looking at the board, looking down, turning head/turning around, talking, standing up, raising hands, and lying on the table. Figure 4 depicts the dataset’s creation process [37].

**Figure 5:**
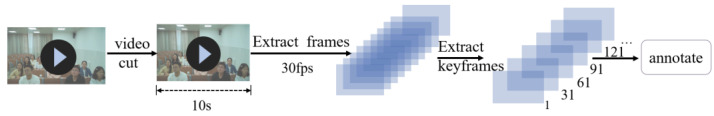
Schematic diagram of the process of extracting key frames from the dataset.

Step 1: Video frame extraction. As shown in Figure 5, the videos were first filtered and cut into videos of 10 s in length for easy labeling, and then the cut videos were divided into frames according to the frame rate of 30 frames per second [37].

Step 2: Extract keyframes. One frame out of every 30 frames per second was extracted as a key frame for that frame, which was used to label student position and student classroom behaviors.

Step 3: Annotate student locations. The extracted keyframes were input into the detector, and the Faster RCNN was employed to detect the students in the keyframes, and the detected student location information was stored in the txt file.

Step 4: Annotate student actions. Due to the characteristics of the time-oriented student classroom behavior dataset, the VIA annotation tool was selected for the multi-label annotation of student behaviors. The txt file results obtained from the detector were converted into JSON data format, and the VIA annotation tool was used to fine-tune the student detection boxes and annotate the classroom behaviors. Finally, an annotation file in AVA format was generated.

**Figure 6:**
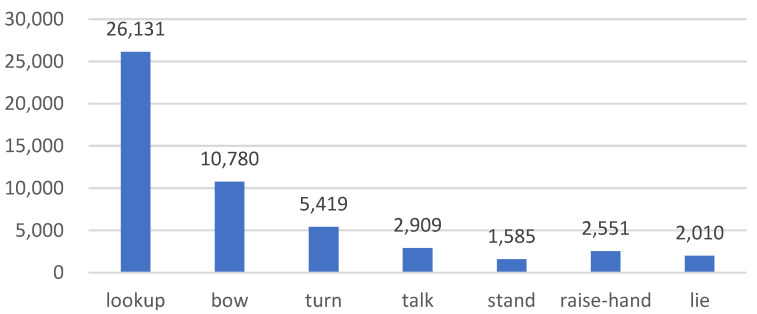
Schematic diagram of the number of various types of behaviors in the dataset.

**Figure 7:**
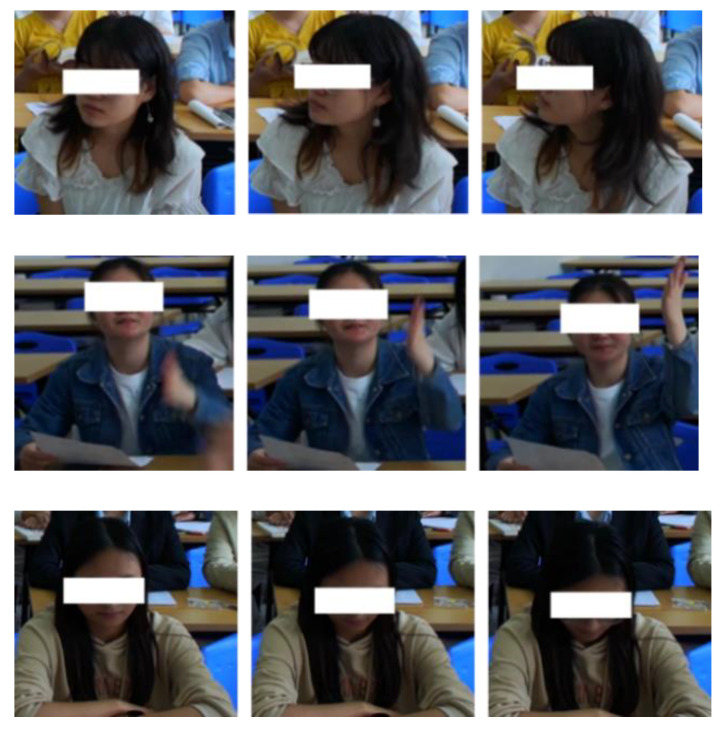
Schematic diagram of the behavioral process of turning their head, raising hands, and bowing.

The total annotation of the final dataset is 51,387. The dataset contains seven kinds of common student actions in the classroom environment, which can reflect the students’ behavior in classroom scenarios. Figure 6 displays the number of labeled categories. Figure 7 depicts the dataset’s head-turning, hand-raising, and head-lowering behavioral processes.

### 4.2. Evaluation Indicators

In this study, the evaluation measures for classroom behavior detection tasks include *Precision*, *Recall*, and *mAP*. The formulae are as follows:

(7) $$Recall=\frac{TP}{TP+FN}\;$$

(8) $$\mathrm{Precision}=\frac{TP}{TP+FN}\;$$

(9) $$mAP=\int_{0}^{1}P\left(R\right)dR\;$$

where TP indicates that both the behavioral class and the predicted behavioral class are positive samples. FP indicates that the true behavioral class is negative, but the predicted behavioral class is positive. FN is an example where the true value of the behavioral class is positive, but the predicted behavioral class is negative.

### 4.3. Ablation Experiments and Analysis

The MSTA and ETA modules introduced in this paper can significantly enhance the algorithm’s ability to detect behavior. Each enhanced module is chosen for ablation experiments in order to test the efficacy of the improved approach presented in this work. A pre-trained model was used in the experiments, and the MSTA and ETA modules are added sequentially to the original SlowFast while retaining the same experimental setup in order to assess each module’s impact on improvement. Table 1 displays the results. The SlowFast backbone network employs 3D ResNet 50, with α taken as 8 and β as 1/8.

According to the experiment results, mAP improved by 5.03% when MSTA was used compared to the original SlowFast. This shows that by substituting the res5 module for the MSTA module in the Slow pathway, the model is better able to receive spatial information, channel information, and temporal information. The addition of the ETA module to the Fast pathway increased the model mAP by 3.44%, indicating that the method enhances the model’s ability to focus on temporal features by adding a temporal attention mechanism to the Fast pathway. It enhances the model’s ability to recognize changes in the action, increasing model accuracy. After introducing both MSTA and ETA, the models achieved better detection results with a 2.35% improvement in Precision, 3.12% improvement in Recall, and 5.63% improvement in mAP. It indicates that better classroom behavior detection can be achieved by adding MSTA in the Slow pathway and also adding ETA time attention in the Fast pathway.

**Figure 8:**
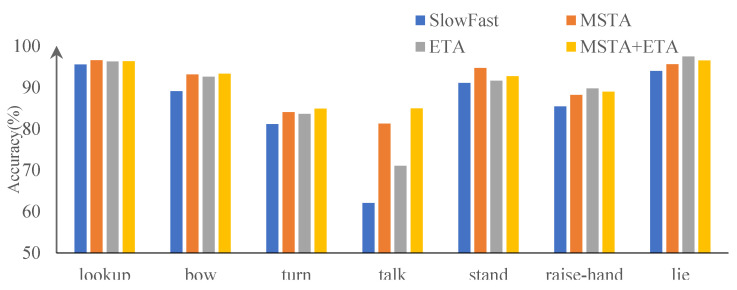
Comparison of behavior category detection accuracy before and after improvement.

The recognition of each behavior type is shown in Table 2 both before and after model modification. It demonstrates that the original algorithm has a superior recognition effect for behaviors with a high sample count (such as looking at the blackboard or lowering your head) and behaviors with more obvious characteristics (such as standing, or lying on a table). However, the accuracy rate of behavior detection with a small sample size and which involved difficulty to distinguish, such as head turning and conversation, was low. The improved model, while maintaining the behavior detection effect with high detection accuracy, greatly improved the detection accuracy of the three behaviors of turning/turning, talking, and raising hands. Figure 8 displays the results of the comparison.

### 4.4. Comparison Experiments and Analysis

A comparative experiment was performed to test the effect on model detection when α was taken to different values. As shown in Table 3, the SlowFast backbone network was taken as 3D ResNet 50, and α was taken as 8 and 4. The experimental results show that both MSTA and ETA made significant improvements on the SlowFast network when α was taken as different values. Additionally, when α was 4, the model detection effect was better, but the model computation was larger due to the number of sampling frames of the Slow path when α was taken as 8. When α was 4, the FLOPs increased by 33.87 and 32.39 G before and after the model improvement, respectively, compared with that when α was 8. The computational effort of the improved model is reduced because MSTA uses grouped convolution for multi-scale spatial feature extraction, which reduces the computational effort, and the ETA does not cause a dramatic increase in computational effort.

**Figure 9:**
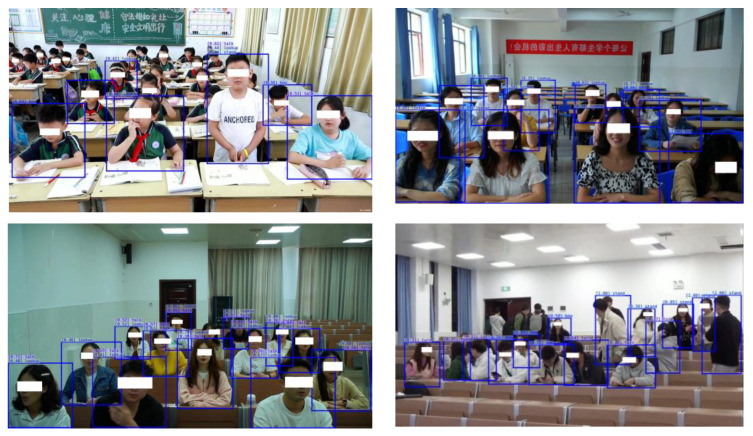
Classroom behavior detection results.

The same number of datasets were utilized under the same configuration conditions to compare the improved SlowFast with the LSTC and Slow-only networks, in order to confirm that it had a better detection effect. The experimental results were mainly evaluated by the mAP evaluation index, and Table 4 displays the precise experiment results. The algorithm used in this paper had an mAP of 91.10% when detecting student behavior in the classroom. Comparing the improved model to SlowOnly and LSTC, it achieved better detection results. This indicates that the improved model performs well in terms of its accuracy in time-oriented classroom behavior detection, and is able to meet the task of detecting students’ classroom behavior in the classroom setting. Figure 9 shows the results of the classroom behavior detection.

## 5. Conclusions

In this paper, we proposed a video classroom behavior detection method based on an improved SlowFast network. To provide model detection accuracy, the attention mechanism was used to improve the network structure. First, MSTA blocks were introduced into the Slow pathway to effectively extract multi-scale spatial information, temporal information and establish long-range channel dependencies. Secondly, the ETA blocks were introduced into the Fast pathway to effectively calculate temporal attention. It was experimentally demonstrated that after the introduction of the two modules, the improved model could achieve a mAP of 91.10% on the self-made student classroom behavior detection dataset, which was 5.63% higher than the original model. It has been shown that the enhanced method suggested in this paper can significantly enhance the model detection effect. The classroom behavior detection requirements using video in a classroom environment can be satisfied using MSTA-SlowFast.

## 6. Discussion

The MSTA-SlowFast model proposed in this paper detects classroom behaviors of instructional video species with practical applications. The analysis of the detected behaviors can be used to achieve the assessment of students’ classroom concentration. Meanwhile, our study can help teachers and school administrators to understand students’ behaviors in time for intervention and management.

Compared with existing studies related to classroom behavior detection, our work implements video-based classroom behavior detection and creates a spatio-temporal-oriented classroom behavior detection dataset. However, our study still has shortcomings. Since SlowFast is implemented using 3D CNN convolution, its detection speed needs to be improved. Moreover, classroom behavior detection is not satisfactory when the video species is more rear-rowed and heavily occluded. As our next step, we will make improvements toward these two aspects.

## Figures and Tables

**Table 1:** Comparison results before and after MSTA and ETA improvement.

Model	Recall/%	Precision/%	mAP/%
SlowFast	79.12	78.78	85.47
SlowFast + MSTA	81.28	81.78	90.50
SlowFast + ETA	80.41	81.16	88.91
SlowFast + MSTA + ETA	81.47	81.90	91.10

**Table 2:** Improved accuracy before and after behavior category detection.

	Lookup	Bow	Turn	Talk	Stand	Raise-Hand	Lie
SlowFast	95.56	89.09	81.13	62.04	91.07	85.4	93.98
MSTA	96.56	93.11	84.03	81.26	94.71	88.20	95.62
ETA	96.25	92.60	83.57	71.05	91.65	89.77	97.50
MSTA + ETA	96.36	93.33	84.84	84.95	92.69	88.99	96.54

**Table 3:** Results of the SlowFast algorithm before and after improvement for different sizes.

SlowFast α	Improve	Size/MB	Param/10^6^	FLOP(G)	mAP/%
α = 8	Before	128.64	33.66	40.62	85.47
	After	121.49	31.79	39.15	91.10
α = 4	Before	128.64	33.66	74.49	87.62
	After	121.49	31.79	71.54	91.19

**Table 4:** The results of the comparison experiment.

Model	Pre-Training	Size/MB	Param/10^6^	mAP/%
SlowOnly (3D ResNet 50)	kinetics400	121.50	31.8	75.38
SlowOnly (3D ResNet 101)	kinetics400	194.15	50.8	82.84
LSTC	kinetics600	274.23	71.8	86.78
SlowFast (3D ResNet 50)	kinetics600	128.64	33.66	85.47
MSTA-SlowFast	kinetics600	121.49	31.79	91.10

## Data Availability

The data presented in this study are available from the https://github.com/weniu/ClassBehavior/ (accessed on 10 May 2023).
